# Improved shear wave motion detection using coded excitation for transient elastography

**DOI:** 10.1038/srep44483

**Published:** 2017-03-15

**Authors:** Xiao-Nian He, Xian-Fen Diao, Hao-Ming Lin, Xin-Yu Zhang, Yuan-Yuan Shen, Si-Ping Chen, Zheng-Di Qin, Xin Chen

**Affiliations:** 1 School of Biomedical Engineering, Shenzhen University, Shenzhen, China; 2 National-Regional Key Technology Engineering Laboratory for Medical Ultrasound, Shenzhen, China; 3 Guangdong Key Laboratory for Biomedical Measurements and Ultrasound Imaging, Shenzhen, China.

## Abstract

Transient elastography (TE) is well adapted for use in studying liver elasticity. However, because the shear wave motion signal is extracted from the ultrasound signal, the weak ultrasound signal can significantly deteriorate the shear wave motion tracking process and make it challenging to detect the shear wave motion in a severe noise environment, such as within deep tissues and within obese patients. This paper, therefore, investigated the feasibility of implementing coded excitation in TE for shear wave detection, with the hypothesis that coded ultrasound signals can provide robustness to weak ultrasound signals compared with traditional short pulse. The Barker 7, Barker 13, and short pulse were used for detecting the shear wave in the TE application. Two phantom experiments and one *in vitro* liver experiment were done to explore the performances of the coded excitation in TE measurement. The results show that both coded pulses outperform the short pulse by providing superior shear wave signal-to-noise ratios (SNR), robust shear wave speed measurement, and higher penetration intensity. In conclusion, this study proved the feasibility of applying coded excitation in shear wave detection for TE application. The proposed method has the potential to facilitate robust shear elasticity measurements of tissue.

Important tissue parameters such as elasticity can be deduced from the study of the propagation of shear waves which can be induced by an external mechanical vibration^1^, acoustic radiation force^2^ and intrinsic physiological motions^3^. Different shear wave elastography techniques utilize different shear wave post-processing techniques to recover tissue mechanical properties. Transient elastography, commercialized as Fibroscan (Echosens, Paris, France), is one of the elastography techniques proposed by Catheline^4^,^5^. It is a one-dimensional quantitative method for assessing the elasticity value of a soft tissue region and has been proven to be useful in numerous clinical applications^6^,^7^,^8^,^9^,^10^. In this method, a transducer which is placed on the axis of the vibrator is used both as a low-frequency piston-like vibrator to generate a transient vibration and as an ultrasonic emitter and receiver to enable monitoring of the propagation of the resulting shear wave in the tissue^11^. The velocity of the shear wave propagation (typically from 1 to 10 m/s) is directly related to the Young’s modulus of the tissue. This technique has the additional advantage that the use of transient excitation prevents a standing wave and other potential artifacts. Its real-time capabilities (typically less than 100 milliseconds) enable it to temporally separate the transmitted elastic wave from the reflected elastic waves. One of the general limitations of TE^12^ is its difficulty in making measurements with obese patients. One reason for this phenomenon is that the intensity of shear wave generated in obese patients is weaker, and another reason is that ultrasonic detection signal suffers more attenuation in adipose tissue^13^. As proposed in previous works^14^,^15^, the shear wave signal SNR is related to the radio frequency (RF) signal SNR. Because the shear wave motion signal is estimated from the ultrasound signal, the weak ultrasound signal can significantly deteriorate the shear wave motion tracking process.

Coded excitation has been used successfully in many fields such radar, communication, and medical ultrasound imaging^16^,^17^. The first investigator that considered the application of coded excitation in medical ultrasound systems was Takeuchi^18^ in a paper dating back to 1979, where the author pointed out the time-bandwidth limitations in the application of coded signals in ultrasound imaging. O’Donnell discussed the expected improvement in SNR, concluding that coded excitation can potentially yield an improvement of 15 to 20 dB^19^. The coding technique utilizes modulated pulse signals with long duration, which can transmit more energy into the tissue without exceeding the maximally allowed peak intensity. This method produces superior ultrasound SNR and penetration depth compared with conventional ultrasound short pulse signals. It has been used for B-mode imaging^20^,^21^,^22^,^23^,^24^,^25^, color flow imaging^26^,^27^, synthetic aperture imaging^28^,^29^,^30^ and ultrasound contrast imaging^31^,^32^,^33^. Coded excitation plane wave imaging for shear wave detection has also been proposed to preserve good penetration and shear wave signal quality^34^. The coded pulse excitation for ultrasonic strain imaging has also been proposed^35^. However, the effectiveness of this technique in TE applications has not been fully studied.

In this work, the coded excitation ultrasound detection system was implemented and successfully used to track the shear wave motion in TE measurement. The Barker 7, Barker 13, and short pulse were used for detecting the shear wave. The potential advantages of such coded signals are increase in robust measurements for the shear wave speed and increase in the SNR for shear wave signals. The penetration depth for TE measurement can also be improved by using the coded excitation.

We organized the article as follows: the implementation of the coded excitation system for TE and experiments designed to evaluate the performance attributes of coded excitation for shear wave detection are described in details in the section of materials and methods. In the results section, two elasticity phantom studies (with and without a pork belly layer) were used to systematically compare the performance of coded excitation and traditional short pulse methods, followed by an *in vitro* liver case study to demonstrate the feasibility of implementing the coding technique in the tissue. The last two sections present discussions and conclusions along with avenues for future research.

## Materials and Methods

### Ultrasound Coded excitation system for TE measurement

**Figure 1:**
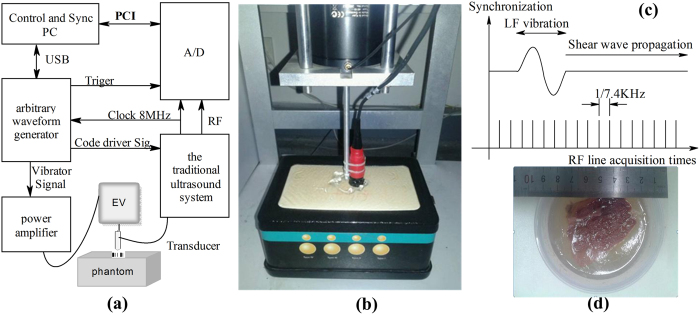
Block diagram of the experimental setup. (**a**) The shear wave generation and coded detection system. (**b**) The experimental setup for TE measurement. (**c**) Acquisition sequence for TE measurement. (**d**) The *in vitro* liver phantom.

A block diagram of the programmable coded excitation system used for TE measurement is shown in Fig. 1(a). A commercial transcranial Doppler ultrasound system (EMS-9UA, Delica, Shenzhen, China) was modified to operate with external excitation. The RF data were acquired using an A/D card (PCI-9846, ADLINK Technology Inc., Taiwan, China). An arbitrary waveform generator (LeCroy Arbstudio 1104, Lecroy Corp. Chestnut Ridge, NY, U.S.) was used to generate the coded excitation signals and the low frequency (LF) vibration signal (50 Hz) with one cycle. The crystal oscillator frequency source (8 MHz) was extracted from the machine and connected to the arbitrary waveform generator as an external clock signal and the A/D card as a sample frequency signal. To generate shear wave motion, a custom vibration system was created, as shown in Fig. 1(b). A single element transducer (center frequency of 2 MHz) was placed on the axis of the electromagnetic vibrator (EV) (Mini shaker Type 4810, Denmark), acting both as a low-frequency piston-like vibrator to generate a transient vibration and as an ultrasonic emitter and receiver to enable monitoring of the propagation of the resulting shear wave in the tissue. The LF signal from signal generator was amplified by a power amplifier (Power Amplifier 2718, Denmark) to drive the vibrator for shear wave generation. A computer was used for overall control of the system.

### Code selection and pulse compression

In this study, the short pulse is 4 sinusoidal cycles at 2 MHz as a chip-waveform. Barker codes with lengths of 7 and 13 were chosen for coding schemes. These codes are optimal in the sense that they have the lowest uniform side-lobe level along the delay axis^20^. The coded pulse sequence was assumed to not be a direct bipolar Barker code of −1 and +1 values but rather a sinusoid at the center frequency of the transducer. Every bit of the Barker code was used to modulate the chip-waveform (where −1 corresponds to a 180 phase shift and +1 corresponds to no phase shift). The duration of the short pulse, Barker 7 and Barker 13 are 2 μs, 14 μs and 26 μs, respectively. The RF signals were collected and compressed with matched filters to recover range resolution^20^. The processing of pulse compression about echo signals is presented as


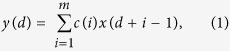


where *d* is the sampling depth in the axial direction, *m* is the sampling length of the code, *x(i*) is the echo signal, and *c(i*) is the corresponding Barker-code sequence for coded transmission.

**Figure 2:**
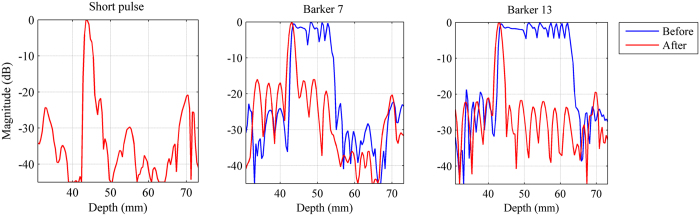
Envelop signals (before and after compression) obtained from a thin wire target for the 3 detection pulse.

Figure 2 shows the echo envelopes (before and after compression) of a thin wire submerged about 43 mm deep in water from the 3 detection pulses. Table 1 shows the summary of the main lobe widths and peak side lobe levels. After pulse compression, the main lobe widths of the coded pulses are comparable with that of the short pulse. The value of the peak side lobe level is close to theoretical value. It should be noted that the processing of pulse compression was performed before the data processing for shear wave motion detection.

### RF signal acquisition and post-processing

**Figure 3:**
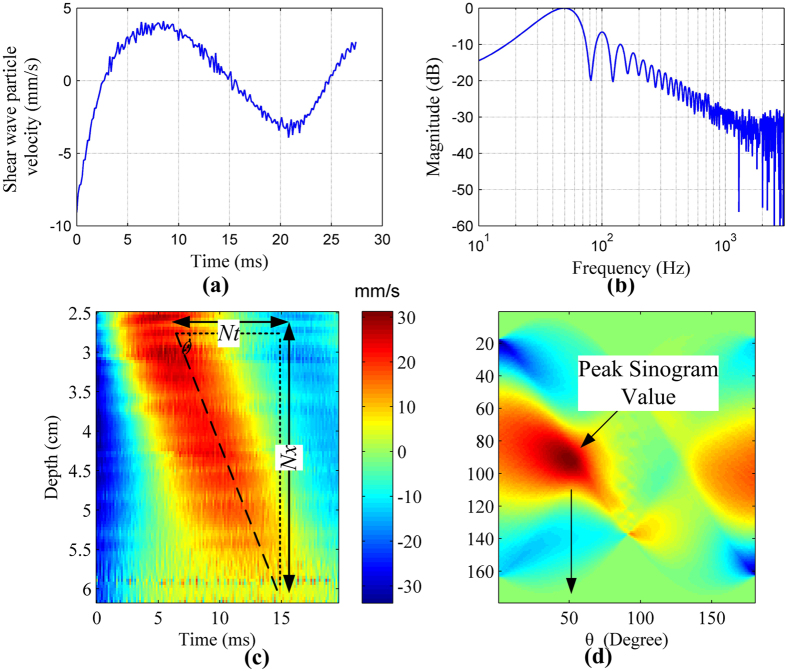
(**a**) The typical shear wave particle velocity signal at a fixed depth. (**b**) The spectrum of the signal in (**a**). (**c**) The shear wave particle velocity image. The black dashed line indicates the shear wave propagation trajectories. N_x_ and N_t_ are the number of pixels of the shear wave motion trajectory along the x and t dimensions, respectively. (**d**) The Radon transform of the shear wave particle velocity image in (**c**). The black arrows indicate the position of the peak sinogram value.

Acquisition sequence for TE measurement is illustrated in Fig. 1(c). The start of acquisition is strictly synchronized with the start of the LF vibration. The RF signals are acquired at a repetition frequency of 7.4 kHz during the propagation of the LF shear wave (50 Hz). After acquisition of the RF data, all post-processing and display were done in MATLAB (version R2008. Mathworks, Inc., U.S.). In our study, a cross-spectrum method proposed by Hasegawa was used to calculate the particle velocity caused by shear wave propagation^36^. Figure 3(a) shows the typical shear wave particle velocity signal at a fixed depth obtained using the short pulse transmission. Two-dimensional particle velocity image was constructed by plotting the signals at different depths into a time-depth space (Fig. 3(c)). A Radon transform was used to convert the particle velocity image into sinogram form in which the shear wave speed (C_s_) can be calculated^37^,^38^,^39^, Fig. 3(d) shows the sinogram of Fig. 3(c). The peak sinogram angle *θ* corresponds to the angle between the shear wave trajectory and the horizontal direction, which is given by


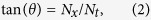


where N_x_ and N_t_ are the number of pixels of the shear wave motion trajectory along the x and t dimensions, respectively. Given that


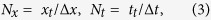


where x_t_ is the actual shear wave propagation distance and t_t_ is the shear wave propagation duration, and Δx and Δt are the pixel sizes along the x and t direction, with


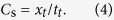


By substituting (2) and (3) into (4), the shear wave speed can be easily derived


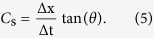


### Phantom experiment

A commercial elasticity phantom (Model 049, CIRS Inc, Norfolk, VA, USA) was used to evaluate the performance of the coded excitation for shear wave detection. This part of the experimental setup is shown in Fig. 1(b). The transducer was placed on the axial of the vibrator, directly touching the top of the commercial phantom. A thin layer of distilled water was poured between the transducer and the phantom surface to ensure good acoustic coupling. The LF excitation central frequency is 50 Hz. The LF excitation input voltage was gradually turned up from 0.1 Vpp to 0.9 Vpp with a 0.2 Vpp interval (5 different values). At each value of the input voltage, the shear wave motion was detected by short pulse, Barker 7, and Barker 13 respectively (3 different detection pulses). Seven acquisitions were taken for each detection pulse at each LF excitation input voltage, and thus a total of 105 data acquisitions were obtained. The positions of the phantom and the ultrasound transducer did not change during these experiments.

### Pork belly phantom experiment

In this experiment, a fresh piece of excised pork belly with a thickness of about 2.0 cm was placed between the transducer and the phantom surface to simulate the body wall. The experiment with pork belly was conducted at room temperature. The pork belly section has clearly delineated layers including skin, muscle, and subcutaneous fat which may introduce more attenuation and ultrasound reverberation clutter noise to shear wave detection. A thin layer of distilled water was poured between the pork belly and the phantom surface to ensure good acoustic coupling. The procedure of the experiment was the same as that of the first experiment and a total of 105 data acquisitions were obtained. This experiment was designed to simulate an *in vivo* situation where RF signal is not strong (i.e. strong ultrasound attenuation).

### *In vitro* liver experiment

This experiment was designed to compare *in vitro* performance of the coded ultrasound signals for TE measurement. As shown in Fig. 1(d), a piece of *in vitro* pig liver with a size of 4 × 5 × 8 cm and depth range from 2–7.6 cm was used for the third experiment. The liver was embedded in 10% agar mixture (by volume). At 0.1 Vpp vibration input voltage, the shear wave motion was detected by short pulse, Barker 7, and Barker 13. One acquisition was taken for each detection pulse at each LF excitation input voltage. A total of 3 data acquisitions were obtained. Both of the pork belly and liver were obtained from the School of Medicine of Shenzhen University. All aspects of the present study were approved by the Animal Care Committee of Shenzhen University and the School of Medicine of Shenzhen University, and all procedures were carried out in accordance with the guidelines issued by the Ethical Committee of Shenzhen University.

### Evaluation Methods

The performances of the coded excitation for TE measurement were systematically evaluated by three parameters: the standard deviation (*SD*) of shear wave speed, the shear wave signal SNR, and the penetration intensity. For each excitation code and each voltage, the mean and *SD* values of shear wave speed were calculated by averaging across the seven repeated acquisitions.

The shear wave signal SNR is given by


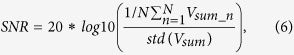


where *N* represents the total number of data acquisition (N = 7),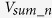 represents the sum of the value along the black dashed line as shown in Fig. 3(c) for the *n*th data acquisition, 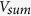 represents the vector data of 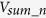 with the length of *N. std* refers to the standard deviation operation. The shear wave signal *SNR* was obtained by calculating the ratio between the mean and standard deviation values of the *V*_*sum*_ measurements.

A quantitative method was proposed to quantify the penetration intensity for the 3 detection pulses. Figure 3(a) shows the typical shear wave particle velocity signal at a fixed depth and Fig. 3(b) shows the spectrum of the signal. The signal energy at about 50 Hz was considered to be the shear wave signal, and the spectrum beyond 1000 Hz was summed and taken as the shear wave detection noise. The penetration intensity at each depth was given by calculating the ratio of the shear wave signal to the detection noise. The penetration intensity plot as a function of depth was then obtained by calculating the penetration intensity for each depth.

## Results

### Phantom experiment

**Figure 4:**
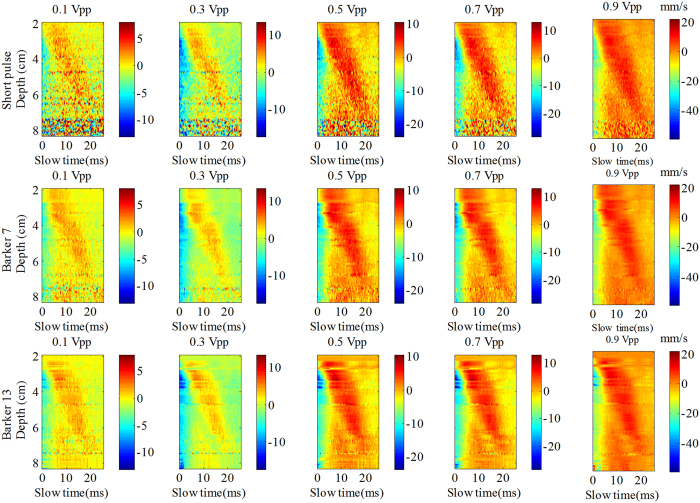
The shear wave particle velocity images obtained by the 3 detection pulses with gradual increase in vibration input voltage from 0.1 Vpp to 0.9 Vpp with a 0.2 Vpp interval. Note the color scale varied according to the different input voltages with units of mm/s.

Figure 4 shows the shear wave particle velocity images for different input vibration voltages and different detection pulses. The color scale was different for the different vibration input voltages. The background noise was clearly visible in the short pulse imaging with the 5 different input voltages while the noise was unnoticeable in the Barker coded excitation imaging. The images obtained by the coded excitation had higher quality than those obtained by the short pulse, with the Barker 13 pulse having the highest quality.

**Figure 5:**
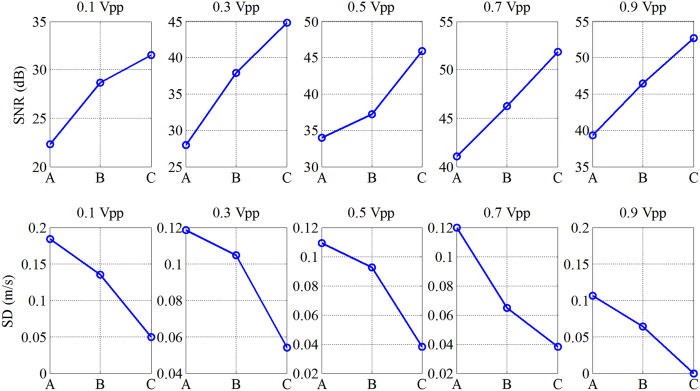
The detailed information about the shear wave signal SNR and the SD values of shear wave speed obtained by the short pulse, Barker 7, and Barker 13 at different vibration input voltages. The letters ‘A’, ‘B’ and ‘C’ were marked in the abscissa represent the short pulse, Barker 7, and Barker 13, respectively.

The detailed information about the shear wave signal SNR and the *SD* value of shear wave speed is plotted in Fig. 5. The letters “A”, “B” and “C” marked in the abscissa represent the short pulse, Barker 7 and Barker 13, respectively. The first row of Fig. 5 shows the shear wave signal SNR with different vibration input voltages for short pulse, Barker 7, and Barker 13. All coded excitation pulses show higher shear wave signal SNR than the short pulse for various vibration input voltages. The SNR of short pulse was 22, 28, 34, 41 and 39 dB at the 5 input voltages, as compared to 32, 44, 46, 52 and 53 dB from the Barker 13. The Barker 13 shows the highest shear wave signal SNR, followed by the Barker 7. The second row of Fig. 5 shows the *SD* values with different vibration input voltages for short pulse, Barker 7, and Barker 13. All coded pulses show lower *SD* value than the short pulse for various vibration input voltages, indicating that the coded excitation provides a more robust shear wave speed measurements than the short pulse. Table 2 lists the mean values of the shear wave speeds detected by the 3 detection pulses. The reported shear wave speed range for the background of the elasticity phantom model 049 is from 2.6 m/s to 3.1 m/s. The mean speed values detected by the various detection pulses were within the reference range.

**Figure 6:**
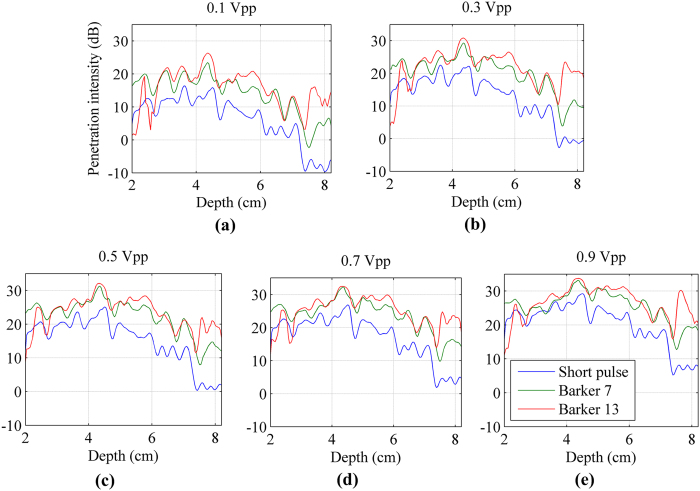
The penetration intensity plots detected by the 3 detection pulses. (**a**) to (**e**) vibration input voltage was gradually increased from 0.1 Vpp to 0.9 Vpp with a 0.2 Vpp interval.

The penetration intensity plots were calculated and plotted as a function of depth for the 3 detection pulses, as shown in Fig. 6. As the vibration input voltage increased, the penetration intensity for all detection pulse increased. At depths shallower than 40 mm, the penetration intensity of coded and non-coded pulses tends to be consistent with the increase of input voltage. However, at deeper depths, the penetration intensity of coded pulses is still higher than the non-coding detection penetration.

### Pork belly phantom experiment

**Figure 7:**
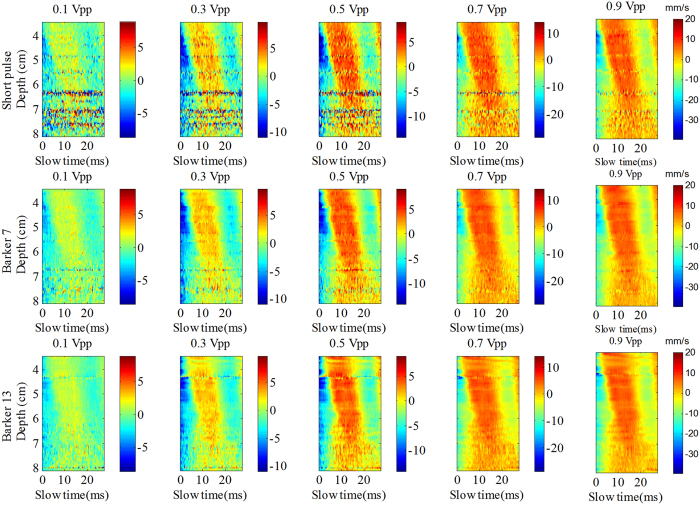
The shear wave particle velocity images after adding the excised piece of pork belly, as obtained by the 3 detection pulses with gradually increasing vibration input voltage from 0.1 Vpp to 0.9 Vpp with a 0.2 Vpp interval. Note the color scale is different for the different input voltages with units of mm/s.

Figure 7 shows the shear wave particle velocity images after adding the excised piece of pork belly, as obtained by the 3 detection pulses with gradually increasing vibration input voltage from 0.1 Vpp to 0.9 Vpp. Compared to the results of the Fig. 4, there is a significant increase in background noise which leads to deterioration of the signal after adding the piece of pork belly. At 0.1 Vpp input voltage, the shear wave particle velocity image obtained by the short pulse becomes rather poor after addition of the pork belly. The shear wave motion trajectory calculated by the coded excitation was better delineated with significantly less background noise than the short pulse. The coded pulse provides better shear wave particle velocity quality than the short pulse, with the Barker 13 pulse having the highest quality.

**Figure 8:**
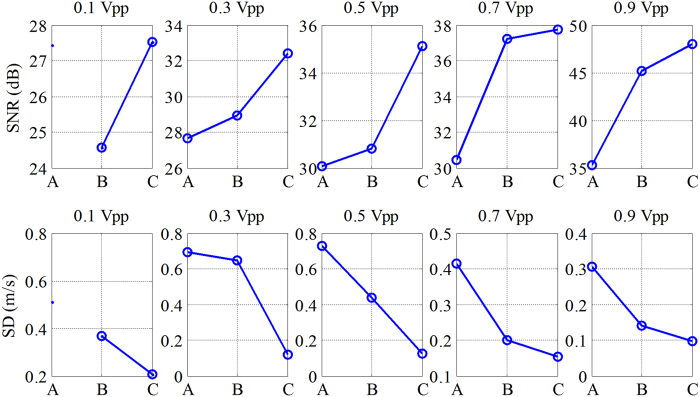
The detailed information about the shear wave signal SNR and the SD values of shear wave speed after adding the excised piece of pork belly, as obtained by the 3 detection pulses at different vibration input voltages. The letters ‘A’, ‘B’ and ‘C’ were marked in the abscissa represent the short pulse, Barker 7, and Barker 13, respectively.

The shear wave signal SNR and the *SD* of shear wave speed for different input vibration voltages and different detection pulses are plotted in Fig. 8. The shear wave signal SNR for the coded pulses was substantially higher than that for the short pulse. The Barker 13 pulse had the highest shear wave signal SNR, followed by Barker 7 pulse. All coded pulses had lower *SD* value than the short pulse for various vibration input voltages. At 0.1 Vpp input voltage, after adding the pork belly, the shear wave particle velocity image detected by the short pulse deteriorates and the shear wave speed measurement becomes incorrect. Therefore, the values of SNR and *SD* for short pulse were not given in Fig. 8. In Table 3, the mean speed values detected by the 3 detection pulses were summarized. The mean speed values detected by the Barker codes were within the reference range while the short pulse was not, especially under the condition with 0.1 Vpp input vibration voltage.

**Figure 9:**
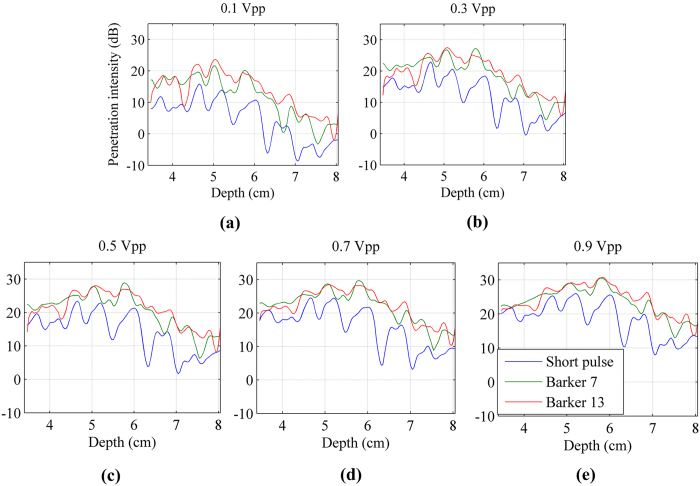
The penetration intensity plots detected by the 3 detection pulses after adding the excised piece of pork belly. (**a**) to (**e**) vibration input voltage was gradually increased from 0.1 Vpp to 0.9 Vpp with a 0.2 Vpp interval.

The penetration intensity plots after adding the excised piece of pork belly were calculated and shown in Fig. 9. The penetration intensity of coded and non-coded pulses tends to be consistent as the input voltage increased at depths shallower than 50 mm. However, at deeper depths (in the noise environment), the penetration intensity of coded pulses are still higher than that of the non-coding detection.

### *In vitro* liver experiment

For the *in vitro* liver experiment, the phantom was made by agar with embedded liver sample. This is a conventional method to produce a lab-made phantom. The quality of the phantom is not as robust as the commercial product. A slight deformation will occur on the surface of phantom after dozens of TE measurements. This deformation may induce measurement bias in the following measurement. Therefore, we only did a limited number of experiments on the liver phantom.

**Figure 10:**
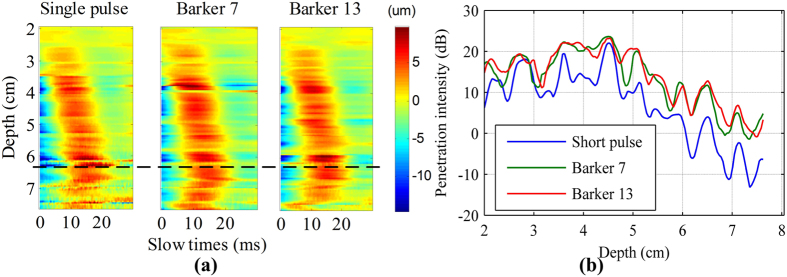
The *in vitro* liver experiment results. (**a**) The shear wave motion signal obtained by the 3 detection pulses at 0.1 Vpp vibration input voltage. (**b**) The penetration intensity plots were calculated from (**a**).

Figure 10(a) shows the shear wave motion signal detected by the 3 detection pulses at 0.1 Vpp vibration input voltage. The coded pulse could consistently detect discernible shear wave motions at low vibration input voltage, while the short pulse failed detection beyond 6.5 cm, as shown in Fig. 10(a). The tissue motion in the deep place was so small that the short pulse was unable to detect this, whereas the coded pulse showed better performance because of its better penetration than the short pulse.

In the liver experiment, the penetration intensity plots were shown in Fig. 10(b). While using the coding method, there was a 5 to 10 mm depth improvement if the penetration depth is defined at the place in the liver where the penetration intensity is lower than 5 dB.

## Discussion

This study investigated the feasibility of implementing coded excitation in TE application for shear wave detection, with the hypothesis that coded ultrasound signals can provide robustness to weak ultrasound signals and good shear wave signal SNR compared with the traditional short pulse. The Barker 7, Barker 13, and short pulse were used for detecting the shear wave in the TE application. The performances of coded excitation were quantitatively compared in terms of the standard deviation (*SD*) of shear wave speed, the shear wave signal SNR, and the penetration intensity. Results from the experiment using a piece of pork belly further demonstrated that coded pulse could consistently track the shear wave motion and provide robust shear wave speed estimates, and this was not achievable using the short pulse under severe noise conditions. Results from the *in vitro* liver experiment study proves the feasibility of implementing the coding technique in the tissue and shows that coded pulses could provide higher robust shear wave signal detection than the short pulse.

One advantage of coded excitation is that it helps to improve the ultrasound signal SNR, which makes it more reliable for shear wave motion detection. These performance improvements can be observed from Figs 4, 5, 7, 8 and 10. One example is shown in Fig. 7 for the piece pork experiment. It is interesting to see that the coded ultrasound signals could still detect the motion well at 0.1 Vpp input voltage, whereas the short pulse could not. This indicates that the shear wave motion still exists but the short pulse ultrasound does not have enough reliability to detect the shear wave motion.

**Figure 11:**
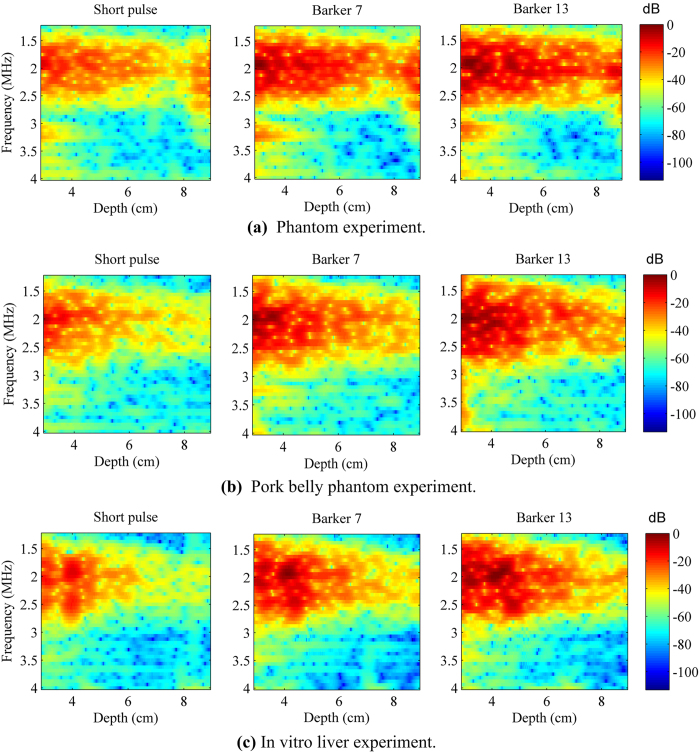
Spectrograms of the 3 detection pulses using short-time Fourier transform in 3 experiments respectively. All spectrograms were normalized to the maximal power of the Barker 13 in the phantom experiment.

Another advantage of coded excitation is that the detection ultrasound signal can penetrate deeper into the tissue and help to improve the detection depth of the shear wave. These performance improvements can be observed from Figs 6, 9 and 10. Figure 11 shows three experiments for the spectrograms of the three detection pulses using a short time Fourier transform on a single A-line. Coded excitation provides greater average power delivered to tissues by increasing the pulse duration and the spectrogram energy was attenuated with increasing depth. Based on the comparison of Fig. 11(a) and (b) under the same color bar and depth range, we found that the power spectrum with pork belly was smaller compared with that without pork belly.

**Figure 12:**
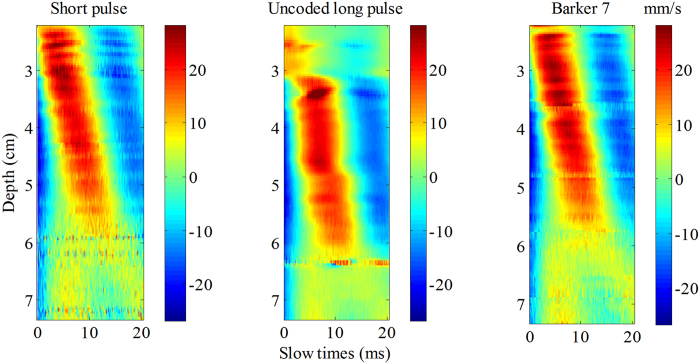
Severe distortion of the shear wave trajectory occurred with non-coded long pulse, while the coded pulse is consistent with the trajectory obtained by the traditional short pulse. Notice that both non-coded long pulse and Barker code pulse have the same pulse duration.

The third advantage of coded excitation is that it can improve the shear wave SNR without compromising axial resolution. Compared with the short pulse, the coded excitation has longer duration and transmits more energy into the tissue to get better SNR and deeper penetration. However, if a non-coded long pulse is used, the axial resolution will be degraded because of the tradeoff between resolution and pulse length^16^,^20^. To demonstrate this advantage of coded excitation, a comparison experiment was conducted between the non-coded long pulse and the Barker code with the same pulse duration. The experimental results shown in Fig. 12 illustrated that non-coded long pulse improved the SNR but reduced the axial resolution while the coded pulse improved the SNR without compromising axial resolution. Poor axial resolution caused severe distortion of the shear wave trajectory in the non-coded long pulse. The trajectory of the shear wave detected by coded pulse is consistent with the trajectory detected by the traditional short pulse. The reconstructed shear wave speeds of elasticity phantom were 2.89 m/s, 4.78 m/s and 2.84 m/s respectively for the 3 detection pulses, short pulse, non-coded long pulse and Barker 13. The speeds value of 4.78 m/s is obviously incorrect which demonstrated that the non-coded long pulse affects the estimation of shear wave speeds.

The coded excitation has its own limitations. The ability to detect near field targets would be decreased, since the minimum distance between the transducer and targeted tissue is determined by the coded pulse duration^25^. One can also see that the selected region of interest was beyond 20 mm depth in all the experiments, as shown in Fig. 4. This is because the duration of the Barker 13 pulse was about 26 μs, which corresponds to about 20 mm of near field.

The coded excitation increases the transmitted power which may cause safety consideration. The FDA regulatory limit for adult diagnostic imaging is 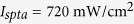 (spatial-peak-temporal-average)^40^. The intensity value of *I*_*spta*_ for conventional short pulse is typically well below 50 Mw/cm^2^ at the focal point^23^. Although the ultrasound safety parameter *I*_*spta*_ is an integration over time and is increased when the duration of the pulse is increased, the intensity value can be increased up to 100–400 mW/cm^2^ by coded excitation, it is still below FDA regulatory limits^16^,^34^.

There is a limitation in this study: local maxima and minima are visible in the penetration intensity plots, especially as shown in Fig. 10(b). This phenomenon presented in Fig. 10(b) is similar to that observed by Catheline *et al*.^5^. Because of the weight of the piston, the real displacement is a damped sinusoid with a low frequency, which caused these displacements to propagate as a compressional wave and as a shear wave. The shear wave component can be interefered with the low frequency compressional wave, and then the amplitude of the shear wave exhibits local maxima and minima. Nevertheless, this did not affect the study of implementing coded technique in TE for shear wave detection. The shear wave dominates, and the overall trend of the shear wave propagation shows negligible interference effects. Use of a lightweight transducer will reduce the influence of the low frequency compression wave and reduce fluctuations in shear wave propagation. Future studies are needed to reduce the mass of the transducer.

The performance of the coded pulses can be further improved. As shown in Fig. 11(c), it can be concluded that the high frequency components of the pulses attenuate quicker than the low frequency. The decoding filters used in this study did not account for such frequency dependent attenuation. Therefore, by designing decoding filters that account for frequency dependent attenuation^41^, the coded pulses performance can be improved. In practice, due to unknown tissue attenuation, it can be challenging to adaptively design depth-dependent decoding filters^34^. Future studies are needed to investigate the impact of depth-dependent filtering on shear wave detection with coded excitation.

In our study, we only considered the Barker 7 and Barker 13 pulses for TE detection. Alternative coding methods, such as chirp code, pseudo-random code, or Golay codes can also be used to track the shear wave propagation. The same coding principle can also be applied to other types of ultrasound probes such as curved and linear arrays for 2-D range of elasticity imaging applications. These will be considered in our future work.

## Conclusions

This paper demonstrated the implementation of coded excitation in TE application for shear wave detection. The Barker 7, Barker 13, and short pulse were used for detecting the shear wave in the TE application. Coded excitation outperformed the short pulse by providing superior shear wave signal SNR, robust shear wave speed measurement, and higher penetration intensity. The experimental results prove the feasibility of implementing coded excitation in shear wave detection for TE applications.

## Additional Information

**How to cite this article:** He, X.-N. *et al*. Improved shear wave motion detection using coded excitation for transient elastography. *Sci. Rep.* **7**, 44483; doi: 10.1038/srep44483 (2017).

**Publisher's note:** Springer Nature remains neutral with regard to jurisdictional claims in published maps and institutional affiliations.

## Figures and Tables

**Table 1:** The summary of the main lobe widths and peak side lobe levels.

Different detection methods	Parameters		
	the main lobe widths	peak side lobe levels	Theoretical peak side lobe levels
Short pulse	1.75 mm	—	—
Barker 7	1.74 mm	−16.58 dB	−16.90 dB
Barker 13	1.87 mm	−21.93 dB	−22.27 dB

**Table 2:** Summary of the mean speed values detected by various methods with different input vibration voltage in the phantom experiment.

Various detection methods	Input vibration voltage				
	0.1 Vpp	0.3 Vpp	0.5 Vpp	0.7 Vpp	0.9 Vpp
Short pulse	2.91	2.78	2.77	2.88	2.92
Barker 7	2.80	2.87	2.74	2.71	2.69
Barker 13	2.92	2.89	2.90	2.94	2.95

The unit of the speed is m/s.

**Table 3:** Summary of the mean speed values detected by various methods with different input vibration voltage in the Pork belly phantom study.

Various detection methods	Input vibration voltage				
	0.1 Vpp	0.3 Vpp	0.5 Vpp	0.7 Vpp	0.9 Vpp
Short pulse	0.94	2.84	2.92	2.86	3.04
Barker 7	2.93	2.92	2.77	2.92	2.89
Barker 13	2.61	2.81	2.89	2.92	2.95

The unit of the speed is m/s.
